# Careful Cheating: People Cheat Groups Rather than Individuals

**DOI:** 10.3389/fpsyg.2016.00371

**Published:** 2016-03-30

**Authors:** Amitai Amir, Tehila Kogut, Yoella Bereby-Meyer

**Affiliations:** ^1^Department of Psychology, Ben-Gurion University of the NegevBeer Sheva, Israel; ^2^Department of Education, Ben-Gurion University of the NegevBeer Sheva, Israel

**Keywords:** ethics, morality, dishonesty, the singularity effect

## Abstract

Cheating for material gain is a destructive phenomenon in any society. We examine the extent to which people care about the victims of their unethical behavior—be they a group of people or an individual—and whether they are sensitive to the degree of harm or cost that they cause to these victims. The results of three studies suggest that when a group (rather than a single individual) is the victim of one’s behavior, the incidence of cheating increases only if the harm to the group is presented in global terms—such that the cheating might be justified by the relatively minor harm caused to each individual in the group (Studies #1 and #3). However, when the harm or cost to each individual in the group is made explicit, the tendency to cheat the group is no longer apparent and the tendency to cheat increases when the harm caused is minor—regardless of whether the victim is an individual or a group of people (Study #2). Individual differences in rational and intuitive thinking appear to play different roles in the decision to cheat different type of opponents: individual opponents seem to trigger the subject’s intuitive thinking which restrains the urge to cheat, whereas groups of opponents seem to trigger the subject’s rational mode of thinking which encourage cheating.

## Introduction

Imagine a contractor who purchased all materials needed for a renovation job he is about to begin. When is he more likely to cheat about the cost of the materials and claim they costed more than what he actually paid: when the homeowner is a single person, or when the work is done for a condominium with several families?

In the present study, we set out to examine whether people care about who bears the consequences of their unethical behavior, and whether the degree of harm they cause when acting unethically plays any part in their decision. Specifically, we compare the action of deceiving a group of people as opposed to a single individual, and gage whether varying the degree of harm caused has any effect on people’s behavior—either when presented in global terms, or when the respective harm to each individual in the group is stated explicitly.

People often engage in dishonest behaviors for material gain (Lewicki et al., 1997; Brief et al., 2001). However, research in the past decade consistently shows that people cheat only to the extent that they can maintain a self-concept of integrity (e.g., Mazar et al., 2008; Ayal and Gino, 2011). Thus, they are more likely to cheat when they feel they can justify their behavior, and the degree of cheating depends on the extent to which they can justify it to themselves (e.g., Shalvi et al., 2011).

However, little research has been done on the effect of the *identity* of the victim of unethical behavior on the tendency to cheat. Gneezy (2005) found that participants in his study were less likely to use deception to increase their payoffs at someone else’s expense. However, in a competitive environment, where participants felt vulnerable in relation to their opponents, they *were* inclined to cheat, as this appears to have provided them with a strong justification to do so (Atanasov and Dana, 2011).

In many cases, it is easier to justify cheating a group by thinking that the harm caused would be distributed among several people, rather than borne by a single individual. Recently, Kesternich et al. (2014) analyzed distributional preferences in games in which decision makers choose the provision of a good that benefits a receiver and creates costs for a group of payers. They found that participants take into account the welfare of all parties and has concerns for efficiency. However, they attach similar weights to small and large groups of players alike, and tend to ignore large costs to the other party when these are shared by many individuals.

Cognitive research of people’s perceptions of single individuals and groups (e.g., Hamilton and Sherman, 1996; Susskind et al., 1999) suggests that a single individual—in contrast to a group of individuals—is viewed as a psychologically coherent unit, which triggers a more extensive processing of information and active integration of the information in real time. As a result, people tend to be more emphatic in their assessment of an individual than of a group, and respond more quickly and confidently when asked to make a judgment about them. In contrast, the comparatively indistinct image of a group makes it easier for subjects to remain detached from it, and thereby easier to deceive for one’s own benefit.

Research on pro-social decision making has confirmed that an individual victim elicits greater empathy and help than a group of victims in the same circumstances (e.g., Small and Loewenstein, 2003; Kogut and Ritov, 2005). Slovic (2007) suggests that this is because it is easier for people to put themselves in the shoes of one person than in the shoes of many. In addition, decisions about groups are expected to be more rational (i.e., take into account “objective” considerations), while decisions about individual victims are expected to be governed more by emotions (Kogut, 2011).

Given the global, more impersonal perception of the group, and the possibility of justifying one’s behavior by the comparatively lesser harm inflicted on each individual in a group, we predicted that participants would tend to cheat a group of opponents more often than a single individual. Furthermore we examined whether informing participants of the specific harm or cost caused to each individual in a group of opponents would attenuate the tendency to cheat the group more than the individual.

To test these hypotheses, we conducted three studies, in which participants were asked to make private predictions of the outcomes of a series of coin tosses while playing against a single opponent or a group of four opponents, and receive payments according to the accuracy of their predictions (Zimerman et al., 2014). Since only the participants knew if their predictions were accurate, this task enabled them to earn more money by giving false reports of their predictions. Since we did not monitor the actual outcomes, we could not determine at the individual level whether or not a participant lied about their predictions, but we could compare their performance on an aggregate basis to that predicted by chance (see Batson et al., 1997; Shalvi et al., 2012; Fischbacher and Föllmi-Heusi, 2013).

In the present study, each correct prediction credited the player with a fixed amount of money—but unlike the above studies, these credits came at the expense of the earnings of an opponent, who was either a single individual or a group. In addition, while the amount earned by the players for each reported correct prediction remained constant in all conditions, the attendant cost to the opponent was varied (either *High* or *Low*). This enables us to examine exclusively the effect of the damage causes to the opponent on the tendency to cheat. In Study #1, the participant was informed only of the overall cost to the opponent group of his or her deception, without reference to the cost to each individual in it; in Study #2, they were informed of the cost incurred by each individual in the group; Study #3 included a direct comparison between the three conditions: a single opponent and the two group conditions that were examined in Studies #1 and #2, (i.e., with and without explicit information regarding the cost to each individual in the group).

## Study #1

### Method

One hundred and forty two undergraduate students (69 of whom were women, *M* = 25.24; *SD* = 3.96) were invited to participate in a short online experiment, and told that 10% of them would be randomly selected to earn money in accordance with their performance in the experiment.

The experiment involved a short task in which each participant was asked to toss a coin twenty consecutive times, after predicting the outcome in each case: for every correct prediction they made, they would earn a fixed amount of money, at the expense of their opponent’s account (which would start with a particular amount). After each coin flip, they were asked to note the outcome on a separate screen, and indicate whether their prediction was correct or not.

Participants were randomly assigned to one of four experimental conditions of a 2 × 2 between-subject design involving two variables: *Cost to Opponent* (being either *high*—NIS 2.0 ∼ USD 0.50), or *low* (NIS 0.50 ∼ USD 0.13), and *Opponent Type* (an individual or a group of four people).

Although the Cost to Opponent varied between the *High* and *Low* conditions, the amount earned by the participant was constant in all instances—NIS 2. Thus, if they predicted all 20 tosses correctly, they could potentially earn as much as NIS 40 (∼US $10)—however, this would be at their opponent’s expense. **Table 1** describes the four conditions, in which the Opponent Type (individual or group) and initial balance varied. As can be seen in the table, since the profit earned by the player remained constant in all conditions (2 NIS for each correct prediction) we kept the cost for the opponent group equal to the participants’ earnings, either at the group level (a total of NIS 2 per group, i.e., 0.5 for each individual in the group) or at the individual level (NIS 2 per each individual in the group, i.e., a total of NIS 8). The cost in the single opponent condition was adjusted to the costs per each individual in the group, and was either NIS 2 or NIS 0.5 (in the high and the low conditions, respectively). Participants in each condition were informed about the payoffs to the self and to their opponent precisely as reported in the table, without indication of the respective cost to each individual in the group condition.

**Table 1:** The four experimental conditions.

Cost to Opponent	Opponent: One individual	Opponent: Four individuals
Low (NIS 0.5)	Initial amount: NIS10 Deduction for each correct prediction: NIS 0.5	Initial balance: NIS 40 Deduction: NIS 2.0 total from the group as a whole (NIS 0.5 each)
High (NIS 2.0)	Initial amount: NIS 40 Deduction for each correct prediction: NIS 2.0	Initial balance: NIS 160 Deduction: NIS 8.0 from the group as a whole (NIS 2.0 each)

Since research on the singularity effect highlighted the role of emotions and intuition in decisions that favor a specific target, we sought to examine the extent to which individual differences in Intuitive-Experiential and Analytical-Rational thinking are related to the decision to act unethically toward single opponents and toward groups. Hence, at the end of the experiment participants were asked to complete the short version of the Rational-Experiential Inventory questionnaire (REI; Epstein et al., 1996), comprising 10 items that gage individual differences between Intuitive-Experiential and Analytical-Rational thinking. (Cronbach’s Alphas 0.86 and 0.82 for the Analytical-rational and the Intuitive-experiential scales, respectively; the correlation between the two scales was not significant (*r* = 0.10, *p* = 0.30).

### Results and Discussion

Means (SDs) of the reported correct predictions are reported in **Table 2**. Overall, participants reported 11.17 correct predictions (*SD* = 2.37)—a significantly higher outcome than the expected chance performance rate (10); *t*(141) = 5.88, *p* < 0.001. This held true for both the *Individual Opponent* condition [10.82, *t*(67) = 2.71, *p* = 0.009] and the *Group Opponent* condition [11.49, *t*(73) = 5.8, *p* < 0.001].

**Table 2:** Mean (SDs) number of reported correct coin toss predictions, in each of the four conditions (Study #1).

Cost to Opponent	Individual Opponent	Group of 4 opponents	Total
Low (NIS 0.5)	*M* = 10.72	*M* = 11.42	*M* = 11.09
	(*SD* = 2.67)	(*SD* = 2.29)	(*SD* = 2.49)
High (NIS 2.0)	*M* = 10.91	*M* = 11.55	*M* = 11.25
	(*SD* = 2.38)	(*SD* = 2.14)	(*SD* = 2.26)
Total	*M* = 10.82	*M* = 11.49	
	(*SD* = 2.51)	(*SD* = 2.20)	

**FIGURE 1:**
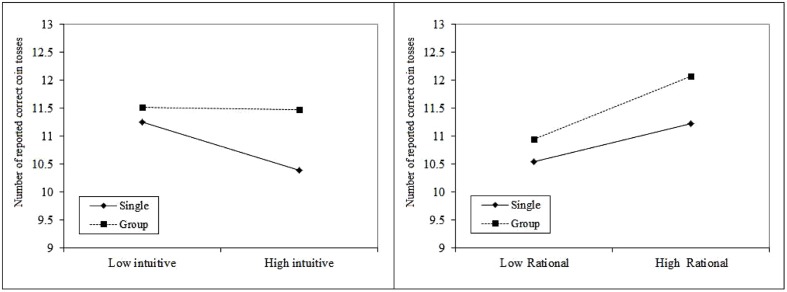
**Mean number of reported correct predictions, as a function of Opponent Type and the two REI sub-scales—plotted as recommended by Aiken and West (1991) and Dawson and Richter (2006), one SD below and one SD above the mean of each subscale in each of the Opponent Type conditions**.

To examine the role played by Opponent Type (individual or group), of the Cost to Opponent (*High* or *Low*), and of the two REI subscales (intuitive-experiential and analytical-rational thinking) in predicting the number of correct coin-tosses reported by the participants (0–20), we conducted a multiple regression analysis. As is recommended for binomial distributions, we performed an ARCSINE transformation of the proportion of correct predictions. The predictors included all four main effects, all two-way interactions and three-way interactions between these variables (see **Table 3**). The overall explained variance of the model was significant *F*(11,130) = 1.92, *p* = 0.04, *R*^2^ = 0.14. The Opponent Type variable made a significant unique contribution to the model (*B* = -3.53, *t* = -2.22, *p* = 0.028). As expected, participants in the Group condition reported more correct predictions (*M* = 11.49) than those in the Individual Opponent condition (*M* = 10.82)—indicating a higher tendency to false reporting when the opponent was a group. In addition, both the interaction between Opponent Type and the Intuitive-Experiential subscale (*B* = 0.96, *t* = 2.27, *p* = 0.025) and between Opponent Type and the Analytical-Rational subscale (*B* = 0.89, *t* = 2.21, *p* = 0.029) were significant. These were plotted in **Figure 1** (right and left, respectively), as recommended by Aiken and West (1991) and Dawson and Richter (2006)^1^. As it demonstrates, when the opponent was a single individual, Intuitive-Experiential ratings correlated negatively with correct predictions, the higher the Intuitive-Experiential tendency the lower the tendency to cheat. In addition, higher Analytical-Rational ratings were linked to increased incidence of cheating when the opponent was a group, and less so when the opponent was an individual. The three-way interaction between Opponent Type and the two Rational-Experiential scales was also significant (*B* = -0.24, *t* = -2.20, *p* = 0.030). No other significant interactions were found: in particular, the Cost to Opponent was not significant (*t* = 0.15, *p* = 0.88), nor did it significantly interact with any of the other variables.

**Table 3:** The regression model – Study #1.

Model	Unstandardized Coefficients		*t*	Significant
	*B*	Standard Error		
(Constant)	1.982	0.300	6.613	0.000
Cost to Opponent	0.164	1.100	0.149	0.882
Opponent Type	-3.531	1.591	-2.219	0.028^∗^
Analytical-Rational	0.034	0.071	0.476	0.635
Intuitive-Experiential	-0.128	0.052	-2.452	0.016^∗^
Opponent ^∗^ Rational	0.889	0.401	2.215	0.029^∗^
Opponent ^∗^ Intuitive	0.957	0.421	2.275	0.025^∗^
Opponent ^∗^ Cost	-0.023	0.096	-0.240	0.811
Cost ^∗^ Rational	-0.144	0.286	-0.505	0.615
Cost ^∗^ Intuitive	-0.100	0.284	-0.352	0.726
Cost ^∗^ Rational ^∗^ Intuitive	0.058	0.075	0.783	0.435
Opponent ^∗^ Rational ^∗^ Intuitive	-0.236	0.107	-2.200	0.030^∗^

*^∗^p ≤ 0.05.*

The results of Study #1 indicate that people tend to cheat a group of opponents more often than an individual one—even when the harm or cost to each individual in the group is the same as that caused to the individual in the Single Opponent condition. Moreover, each condition appears to trigger a different mode of thinking: when faced with an individual opponent, the subject’s Intuitive thinking tends to restrain their urge to cheat, whereas when faced with a group of opponents, the subject’s Rational mode of thinking appears to encourage cheating. These results are in line with research on the *singularity effect*—namely, that a single opponent triggers a spontaneous emotional response in the subject that tends to result in decisions that are more favorable to the opponent (Kogut, 2009).

In the present study participants in the Group condition were informed of the cost of their deception to the group as a whole, without reference to the cost to each individual within the group. Thus, informing participants of the cost incurred by each individual in the opponent group may make people care more about each such individual, thereby making it more difficult for them to use the comparatively minor harm caused to each individual in the group as an excuse for cheating. To test this possibility, in Study #2 we replicated Study #1, but added information about the cost incurred by each group member in the Group Opponent conditions.

## Study #2

Participants: One hundred and fifty two undergraduate students (81 of whom were women—*M* = 24.8, *SD* = 1.89), who were randomly assigned to one of the four experimental conditions of the same 2 × 2 between-subject design as in Study #1. Here too, they were invited to take part in a short online experiment, and told that 10% of them would be randomly selected to earn money according to their performance in the experiment. We used the same method as in Study #1—with one difference: in the Group Opponent condition, the participants were informed of the respective cost of their deception to each individual in the opponent group, as well as the total cost to the group as a whole.

### Results and Discussion

The means (SDs) of the correct predictions reported in each condition are presented in **Table 4**. Overall, participants reported 12.21 correct predictions (*SD* = 2.95)—once again, significantly higher than the expected chance prediction rate (10); *t*(151) = 9.24, *p* < 0.000. This was true for both reports in the Single Opponent condition [12.32, *t*(70) = 6.94, *p* = 0.001] and in the Group condition [12.11, *t*(80) = 6.19, *p* < 0.001].

**Table 4:** Mean number of reported correct predictions (SD), in each of the four conditions (Study #2).

Cost to Opponent	Single Opponent	Group of 4	Total
Low	*M* = 12.97	*M* = 12.47	*M* = 12.69
(0.5 NIS)	(*SD* = 2.94)	(*SD* = 3.11)	(*SD* = 3.09)
High	*M* = 11.69	*M* = 11.71	*M* = 11.70
(2 NIS)	(*SD* = 2.42)	(*SD* = 3.00)	(*SD* = 2.71)
Total	*M* = 12.32	*M* = 12.11	
	(*SD* = 2.82)	(*SD* = 3.01)	

To examine the role of the two independent variables (Opponent Type and Cost to Opponent) in predicting the participants’ reports, we conducted a 2 × 2 ANOVA on the transformed ARCSINE data of the proportion of the correct prediction of coin tosses as a function of the Opponent Type (individual or group) and the Cost to Opponent (High or Low). The results revealed no main effect for the Opponent Type *F*(1,148) = 0.15, (NS). However, the main effect for the Cost to Opponent approached significance [*F*(1,148) = 3.72, *p* = 0.056, $\eta_{p}^{2}$ = 0.025]—in that participants may have reported overall greater success in their predictions under the Low Cost condition (*M* = 12.69) than under the High Cost condition (*M* = 11.70). No significant interaction was found [*F*(1,148) = 0.36, NS].

The results of Study #2 indicate that when the actual cost to each individual in the opponent group is stated explicitly, participants likely care about the harm they may cause to others, irrespective of whether it is a single individual or a group. In these instances, the magnitude of harm or cost caused comes into play: when the harm or cost to each individual in the opponent group is minor, participants are inclined to cheat more often. In other words, informing the participant of the harm caused to each opponent group member appears to increase the participant’s awareness of each individual in the group. It also appears to make it more difficult for the participants to use the relative minor harm to each individual in the group as a pretext for cheating (especially in the High Cost condition).

Taking the results of the two experiments together suggests that when the partner is a group, the extent of cheating depends on the way in which the information about the damage is presented: When the damage appears globally, without specifying the cost to each individual, people tend to cheat a group more than a single opponent; while, when the exact damage caused to each individual in the group is explicitly given, level of cheating groups and single opponents does not significantly differ. However, the two studies do not allow a direct comparison between the two groups (with and without explicit details on the extent of damage caused to each individual in the group), since different samples were examined, which differ in the overall extent of cheating. Hence, we conducted another study with three between subject groups: a single recipient, a global group (in which the damage caused to the group appears globally) and a detailed-group (in which the damage caused to each individual in the group is explicitly specified). In this study we kept the cost to each single opponent (whether an individual, or an individual in a group) constant (always two shekels, which is equal to the amount earned by the participant for each correct report).

## Study #3

Participants: One hundred and nine undergraduate students (56 women—*M* = 25.87, *SD* = 3.97), who were randomly assigned to one of the three experimental between-subject conditions: (1) a single recipient, (2) a group of four recipients with information on the respective cost of a deception to the group as a whole (hereafter “Global-group”), and (3) a group of four recipients with information on the respective cost of a deception to each individual in the opponent group, as well as the total cost to the group as a whole (hereafter “Detailed-group”). Here too, participants were invited to take part in a short online experiment, and told that 10% of them would be randomly selected to earn money according to their performance in the experiment. The method was the same as in the high cost conditions in Studies #1 and #2 in the previous studies (see **Table 5**). In addition, as in Study #1, participants were asked to complete the short version of the Rational-Experiential Inventory questionnaire (REI; Epstein et al., 1996), at the end of the experiment (Cronbach’s Alphas 0.80 and 0.85 for the Analytical-rational and the Intuitive-experiential scales, respectively; the correlation between the two scales was not significant (*r* = -0.07, *p* = 0.41).

**Table 5:** The three experimental conditions – Study #3.

A Single opponent	A global group	A detailed group
Initial amount: NIS 40 Deduction for each correct prediction: NIS 2.0	Initial balance: NIS 160 Deduction: NIS 8.0 from the group as a whole	Initial balance: NIS 160 Deduction: NIS 8.0 from the group (i.e., NIS 2.0 from each individual)

### Results and Discussion

Mean (SDs) number of reported correct coin toss predictions, in each of the three conditions are presented in **Table 6**. Overall, participants reported 11.06 correct predictions (*SD* = 2.69)—a significantly higher outcome than the expected chance performance rate (10); *t*(108) = 4.12, *p* < 0.001. However, in the *Single Opponent* condition, reported correct predictions were not significantly different from the ones expected by chance [10.62, *t*(33) = 1.33, *p* = 0.191]. The difference between reported correct predictions in the *Detailed*-*Group* condition and the expected outcome by chance (10.84) approached significance [*t*(31) = 1.97, *p* < 0.058]; while reports in the *Global*-*Group* condition were significantly different than the expected by chance [11.57, *t*(42) = 3.61, *p* < 0.01].

**Table 6:** Mean (SDs) number of reported correct coin toss predictions, in each of the three conditions (Study #3).

Condition	Mean (*SD*)
Single Opponent	10.62 (2.70)
Detailed-*Group*	10.84 (2.37)
Global-*Group*	11.57 (2.88)

To examine the role played by Condition (Single, Global-group, and Detailed-group), and of the two REI subscales (Intuitive-experiential and Analytical-rational thinking) in predicting the number of correct coin-tosses reported by the participants (0–20), we conducted a multiple regression analysis on the ARCSINE transformation of the proportion of the correct prediction of coin tosses (see **Table 7**). Two dummy variables were created (Single and Detailed) using the Global group condition as the comparison group. Thus, the predictors included all four main effects (the Single and the Detailed-group dummies, the intuitive-experiential and the analytical-rational thinking scales and all two-way interactions between these variables. The overall explained variance of the model was significant *F*(9,99) = 3.62, *p* = 0.001, *R*^2^ = 24.8. The contribution of the Single dummy – comparing the Global-group to the Single opponent conditions was significant (*B* = 0.55, *t* = 2.01, *p* = 0.047); such that participants in the Global-group reported more correct predictions (11.57) than participants in the Single opponent condition (10.62)—indicating a higher tendency to false reporting when the opponent was a Global-group, replicating the results of Study #1. The main effect of the Detailed-group dummy, comparing the Global-group to the Detailed-group, approached significance (*B* = 0.56, *t* = 1.67, *p* = 0.099); such that reported correct predictions were higher in the Global group than in the Detailed group (10.84). In addition, the interaction between the Single dummy and the Analytical-Rational subscale was significant (*B* = -0.24, *t* = -3.07, *p* = 0.003); such that higher Analytical-Rational ratings were linked to increased incidence of cheating only in the Global-group condition; while in the single opponent condition higher Analytical-Rational ratings were liked to a decrease in the number of reported correct predictions. Finally, the interaction between the Intuitive-experiential scale and the Detailed-dummy was significant (*B* = -0.25, *t* = -2.09, *p* = 0.04), showing that intuitive thinking is correlated with higher reports of correct predictions in the Global group condition, and with fewer reports of correct predictions in the Detailed-group condition.

**Table 7:** The regression model – Study #3.

Model	Unstandardized Coefficients		*t*	Significant
	*B*	Standard Error		
(Constant)	1.908	0.425	4.484	0.000
Single	0.549	0.272	2.014	0.047^∗^
Detailed-group	0.563	0.338	1.667	0.099
Rational	-0.198	0.133	-1.489	0.140
Intuitive	-0.170	0.176	-0.967	0.336
Rational × Intuitive	0.120	0.056	2.161	0.033^∗^
Single × Intuitive	-0.025	0.093	-0.269	0.788
Detailed-group × Intuitive	-0.247	0.118	-2.086	0.040^∗^
Single × Rational	-0.245	0.080	-3.074	0.003^∗∗^
Detailed-group × Rational	-0.023	0.087	-0.263	0.793

*^∗^p ≤ 0.05; ^∗∗^p ≤ 0.01.*

In summary, the results of the third study support the conclusions of Studies #1 and #2 by showing that people tend to cheat a group more than a specific individual, mostly when the cost or harm to the group is presented in global terms. However, when the cost to each individual in the group is explicitly given, participants tend to cheat groups and individual opponents to a similar degree. The results also support the idea that higher analytical-rational thinking is related to the tendency to cheat a global group, replicating the results of Study #1. However, the results for the Intuitive-experiential scale were only partially consistent with the results of Study #1 by demonstrating a decrease in reported number of correct predictions in the Detailed-group condition (replicating the direction of results found in Study #1 for single opponents). However, in the global group condition the Intuitive-experiential scale was correlated with higher reports of correct predictions.

## General Discussion

Cheating is a destructive phenomenon in any society. When presented with an opportunity to profit by cheating, most people will do so—but only to a limited extent, to maintain their positive self-image as honest individuals (e.g., Mazar et al., 2008). However, when given a pretext for such behavior, they are more likely to cheat and to profit at the expense of others (e.g., Shalvi et al., 2011). Our results confirm that participants do cheat to some extent when faced with either a group of opponents or an individual one. However, if the consequent cost or harm to the group is presented in global terms, participants tend to cheat more often than when the opponent is an individual—perhaps because they imagine that the cost to each individual in the group is comparatively minor (Studies #1 and #3). Conversely, when they are explicitly told of the cost to each individual in the opponent group, participants tend to heed this and cheat groups and single opponents to the same degree (Studies #2 and #3). Furthermore, in such cases, participants are sensitive to the degree of harm or cost they cause, and tend to cheat more often when the harm they cause is less severe—regardless of whether their opponent is a single person or a group (Study #2). Since the amount of money earned from each correct prediction was the same in all studies, and is easily divisible by four (the number of people in the group), one might expect people to calculate the cost to each individual in the opponent group and show greater sensitivity as a result, even when this information is not explicitly stated. However, the results of our studies suggest that participants act only on the information given to them: when the cost of the deception to the group is presented in global terms, they appear to use the undefined cost to each individual opponent as a pretext for cheating.

As previously noted, research suggests that people perceive groups in a more global and impersonal fashion, which may induce a greater psychological distance and diminish their level of caring (Kogut and Ritov, 2005; Slovic, 2007). However, making the presence of each individual in a group more salient may increase a subject’s level of concern for the group (Bartels and Burnett, 2011). This may have occurred in Studies #2 and #3, when participants were explicitly told of the respective loss that each member of the opponent group would incur as a result of their deception. This finding is in line with the *self-concept maintenance* model put forward by Mazar and Ariely (2006), which states that portraying unethical actions as more offensive may increase the internal cost of engaging in such actions, and discourage people from behaving dishonestly.

According to the standard economic model, individuals aim to maximize their own profit. A “rational” individual is therefore one who chooses the option that is expected to yield them the greatest profit (e.g., Hobbes and Macpherson, 1968; Smith, 1999). From this perspective, a person’s decision to be honest depends only on the expected external benefits and costs to themselves (Lewicki, 1983; Hechter, 1990). The results of Studies #1 and #3—according to which individual differences in Analytical-Rational thinking predict a greater incidence of cheating—are in line with this model. However, this prediction was significant only when the opponent was a global group—not a single individual. The relatively detached perception of the group (compared with the perception of a single individual) allows for more “rational” thinking (as defined by the standard economic model), and appears to increase the incidence of cheating for monetary gain. Interestingly, when the opponent is a single individual (which possibly fosters greater perspective taking—Slovic, 2007), Intuitive-Experiential thinking tends to come into play, resulting in diminished cheating—possibly due to the subject’s greater empathy toward the opponent. This pattern was also found for the Detailed-group, a group-setting that makes the individuals in the group more salient (Study #3). However, we did not find a replication for this direction in the single opponent condition in Study #3, and it is for future research to further explore this issue.

In many real-life situations, the precise harm caused by one’s unethical behavior is not readily apparent. Our results suggest that in such cases people tend to cheat more often when their opponent is a group (as opposed to an individual)—perhaps on the assumption that the harm to each group member is minor compared with the harm that would have been caused to a single individual. Research has revealed various situations in which people fail to notice that their behavior violates their own moral standards (Gino et al., 2010; Bazerman et al., 2011; Schurr et al., 2012). Our research suggests that when considering a cost for a group of people, providing explicit information about the respective harm or cost that it would cause to each individual group member may reduce the incidence of such undesirable behavior, but only in cases when the relative cost to each group member is significant.

## Ethics Statement

The research has been approved by the institutional review board of Ben-Gurion University of the Negev, Israel.

## Author Contributions

All coauthors designed the research. AA conducted first study. The second was conducted by a research assistant. All coauthors analyzed the data. All coauthors participated in writing the paper, which was led by TK.

## Conflict of Interest Statement

The authors declare that the research was conducted in the absence of any commercial or financial relationships that could be construed as a potential conflict of interest.

## Footnotes

1 According to Aiken and West (1991) in order to examine an interaction between two independent variables found in a regression analysis, the simple slopes of the regression should be plotted, using two meaningful points to anchor each line. Theoretically, one could choose any two values within the observed range of the DV to plot each line. It is most common to choose the mean, 1 SD below the mean, and 1 SD above the mean of a continuous variable.
